# Pomological and Biochemical Characterization of Autochthonous Grape Varieties Grown in Türkiye

**DOI:** 10.1002/fsn3.72124

**Published:** 2026-08-04

**Authors:** Yakup Karasu, Adnan Dogan, Semih Aykut, Burhan Ozturk, Erdal Aglar

**Affiliations:** ^1^ Faculty of Agriculture, Department of Horticulture Van Yüzüncü Yıl University Van Türkiye; ^2^ Faculty of Agriculture, Department of Horticulture Ordu University Ordu Türkiye; ^3^ Faculty of Arts, Design and Architecture, Department of Landscape Architecture Munzur University Tunceli Türkiye

**Keywords:** antioxidant activity, autochthonous grape genotypes, multivariate analysis, phenolic compounds, pomological characterization

## Abstract

Grapevine ( *Vitis vinifera* L.) is an important fruit crop with considerable morphological and biochemical diversity, and autochthonous genotypes represent valuable genetic resources for breeding and conservation programs. Many local varieties remain underexplored in terms of pomological and antioxidant traits. Eighteen indigenous grape genotypes collected from Muş province (Eastern Anatolia, Türkiye) were evaluated using morphological and biochemical parameters, including berry traits, phenolic composition, vitamin C, and antioxidant activity. Multivariate analyses including PCA, HCA, and Pearson correlation were performed to classify genotypes and determine trait relationships. The first two PCA components explained 51.9% of the total variation. Antioxidant‐related traits mainly contributed to PC1, whereas berry size and anthocyanin content influenced PC2. HCA grouped the genotypes into distinct clusters according to antioxidant capacity and pomological characteristics. Correlation analysis showed significant positive relationships among antioxidant‐related parameters. Berry size traits were positively correlated with each other, but negatively associated with seed number and skin thickness. The combined use of pomological, biochemical, and multivariate analyses proved useful for genotype classification. The identification of antioxidant‐rich genotypes may support future breeding and conservation studies. The results highlight the importance of indigenous grape germplasm for sustainable viticulture.

## Graphical abstract

Eighteen autochthonous grape genotypes cultivated in Eastern Anatolia (Muş, Türkiye) were comprehensively evaluated for pomological traits, phenolic composition, vitamin C content, and antioxidant capacity. Multivariate analyses (PCA, hierarchical clustering, and correlation) revealed pronounced genotype‐dependent variation and clear trait‐based groupings. Antioxidant‐related parameters (phenolics, flavonoids, DPPH, FRAP) were the primary drivers of genotype discrimination, while berry size and skin color defined morphological differentiation. Genotypes such as Dımışkı, Sulu Üzüm, and Kuru Üzüm exhibited superior antioxidant potential, highlighting indigenous grape germplasm as a valuable resource for functional food development, breeding, and sustainable viticulture.

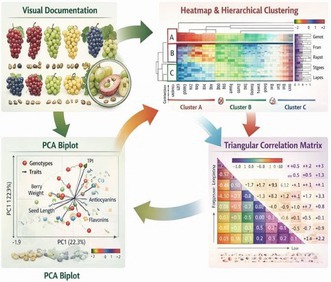

## 1 Introduction

Grapevine ( *Vitis vinifera* L.) is among the most economically and nutritionally important fruit crops worldwide due to its rich biochemical composition and long cultivation history (Celik 2006; Gazioglu Sensoy and Tutus 2017). Türkiye possesses rich grape genetic diversity due to its diverse ecological conditions (Agaoglu and Celik 1985), and Eastern Anatolian regions such as Muş may represent important reservoirs of underexplored autochthonous grape germplasm. According to FAO (2022), grapes are grown on approximately 6.9 million hectares worldwide, with Türkiye ranking fifth in global production after Spain, China, France, and Italy.

Grapes are important sources of phenolics, flavonoids, anthocyanins, and other antioxidant compounds associated with various health‐promoting properties (Xia et al. 2010; Lim 2013; Huang et al. 2017). Most of these phytochemicals are predominantly concentrated in grape skins and seeds, with flavonoids playing a key role in neutralizing free radicals. Anthocyanins, particularly malvidin‐3‐glucoside, are responsible for the red to purple coloration in black grape cultivars and contribute significantly to their antioxidant capacity (Ribéreau‐Gayon et al. 2000; Costa et al. 2014).

The phenolic content and associated antioxidant potential of grapes are highly variable and influenced by genotype, environmental conditions, and cultural practices (Bas 2018; Shahab et al. 2023). Previous studies have demonstrated significant variability in phenolic composition and antioxidant capacity among grape genotypes depending on ecological and genetic factors (Ozden and Vardin 2009). In Turkish cultivars, total phenolic content has been reported to range between 79.2 and 154.6 mg/g, highlighting significant biochemical diversity among genotypes (R. Gazioglu Sensoy 2012). Recent research also emphasizes the role of abscisic acid in regulating anthocyanin biosynthesis via the phenylpropanoid pathway, further influencing berry color and quality (Shahab et al. 2023).

Despite Türkiye's rich grape genetic resources, many autochthonous cultivars remain insufficiently characterized in terms of their biochemical profiles and fruit quality traits.

This study hypothesizes that autochthonous grape genotypes grown in Muş province exhibit significant variation in fruit morphology, phenolic composition, and antioxidant capacity, and that multivariate analysis can reveal meaningful trait‐based groupings and correlations. To test this hypothesis, 18 regionally adapted genotypes were evaluated for their pomological parameters, skin color, seed traits, and biochemical contents including total phenolics, flavonoids, anthocyanins, and antioxidant activity.

By integrating classical pomological evaluation with biochemical assays and multivariate statistical tools (PCA, clustering, correlation), this research aims to contribute to the valorization of Türkiye's autochthonous grape germplasm and support future breeding, conservation, and nutritional evaluation efforts.

## 2 Materials and Methods

### 2.1 Plant Material

This study was conducted during the 2022 harvest season in Muş province, Eastern Anatolia, Türkiye (38.95° N, 41.75° E), a region with a long‐standing tradition of viticulture. The plant materials used in this study consisted of 18 autochthonous grape genotypes collected from local farmers' vineyards located in the central district of Muş. All sampled plants were naturally cultivated and not taken from protected wild populations. The formal identification of each genotype was performed by experts from the Department of Horticulture, Faculty of Agriculture, Van Yüzüncü Yıl University. Voucher specimens were deposited in the Herbarium of Van Yüzüncü Yıl University (VANF) under accession numbers VV‐2022‐001 to VV‐2022‐018. Collection of plant materials was carried out with verbal permissions from vineyard owners, and all procedures complied with national and institutional regulations (Environment Law No. 2872 and Plant Variety Protection Law No. 5042). Field studies were conducted in accordance with local legislation. The area has a continental climate characterized by hot, dry summers and cold, snowy winters. The annual mean temperature is 9.7°C, and the average daily sunshine duration is 6.6 h. Vineyards were located at altitudes between 1200 and 1800 m on sloped terrain, cultivated under dry farming conditions using goblet training systems and organic manure fertilization.

A total of 18 autochthonous grape genotypes were selected for evaluation based on regional cultivation history, morphological diversity, and skin color classification.

According to measured skin color values, the genotypes were grouped as follows:

- Green‐yellow to yellow‐green skinned genotypes: Beyaz Üzüm, Güz Beyazı, Keji Beyazı, Lüle Beyazı, Sinciri 1, Sinciri 2.
- Red to red‐black skinned genotypes: Dımışkı, Elazığ Kırmızısı, Vakkas Dişi.
- Dark red‐purple skinned genotypes: Dana Gözü 1, Dana Gözü 2, Kaşper, Keçi Memesi, Kırmızı Üzüm, Kuru Üzüm, Sulu Üzüm, Vakkas, Vakkas Erkek.

The study was conducted during a single harvest season (2022). For each genotype, samples were collected from three biologically independent vines located in different vineyard positions within the same production area. Each biological replicate consisted of separately harvested grape clusters collected at commercial maturity based on skin color development and soluble solids content. Morphological and biochemical analyses were performed using these biological replicates to minimize sampling bias and improve data reliability. However, since the study was limited to a single growing season and one geographical region, the potential influence of year‐to‐year climatic variability and genotype × environment interactions on grape composition should be considered when interpreting the results.

**FIGURE 1:**
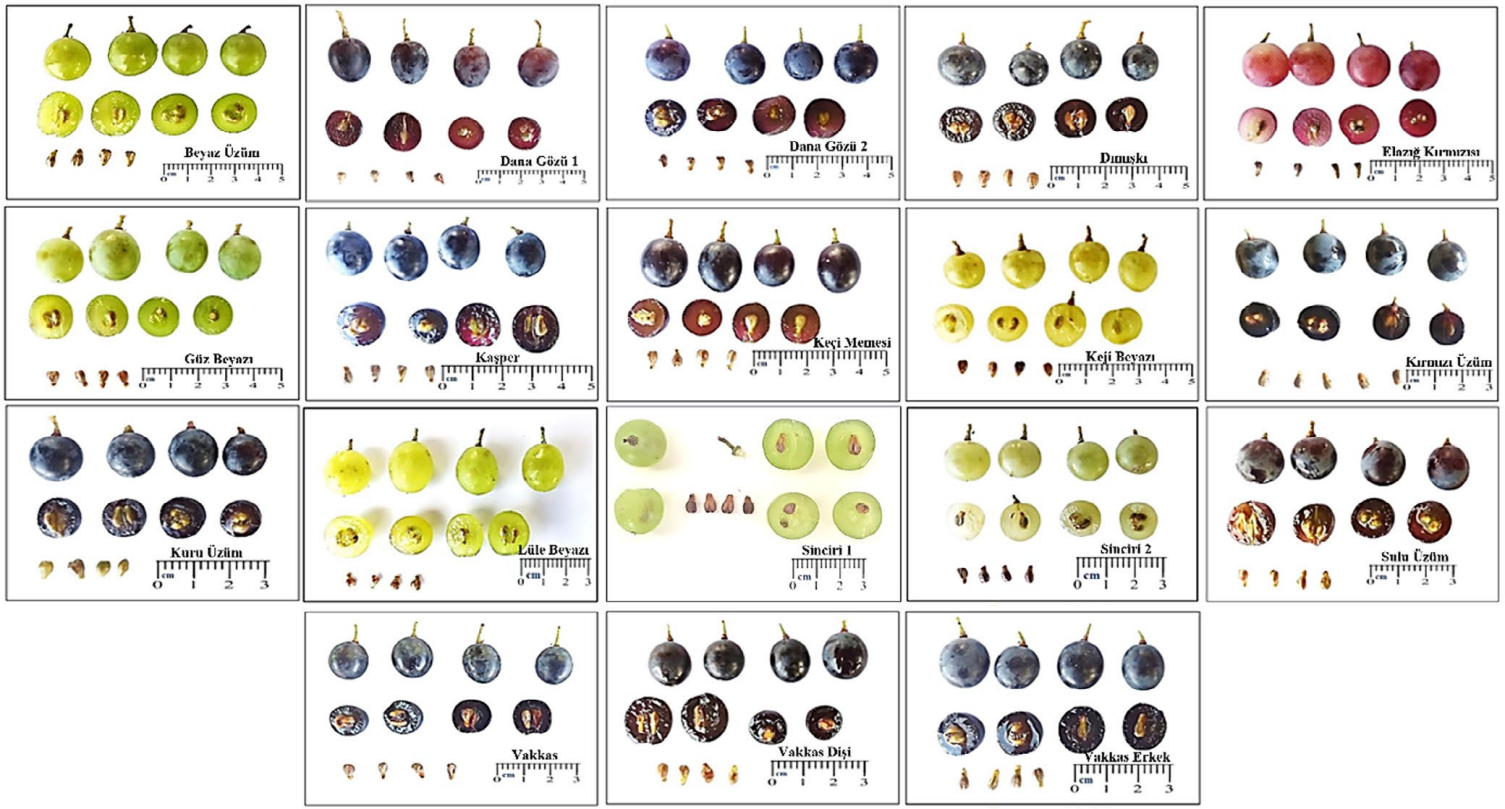
Visual documentation of grape berries and seeds from 18 autochthonous genotypes.

These autochthonous cultivars exhibited significant variation in skin thickness (0.21–0.56 mm), seed number (2.19–3.38), and seed weight (26.21–46.57 mg), supporting their differentiation in pomological and biochemical analyses. Visual documentation of whole berries, cross‐sections, and seeds is presented in Figure 1. Each genotype is represented by whole berries, longitudinal cross‐sections, and extracted seeds. The visual comparison supports pomological differentiation and complements measured traits such as skin color, seed morphology, and berry structure.

### 2.2 Pomological Properties

Thirty berries were randomly selected from each autochthonous grape genotype and weighed using a precision balance with ±0.01 g accuracy. The average berry weight was calculated by dividing the total weight by the number of berries. Berry width was measured at the widest equatorial point, and berry length was measured from the stem end to the opposite tip using a digital caliper with ±0.01 mm accuracy. These procedures were adapted from Dogan and Uyak (2020).

### 2.3 Total Soluble Solids, Titratable Acidity and Vitamin C

A sufficient amount of grape berries was juiced using an electric juicer and filtered through cheesecloth. The juice was analyzed for total soluble solids using a digital refractometer (PAL‐1, Atago, USA), and values were expressed as percentage (%), following standard procedures described in Dogan and Uyak (2020). For titratable acidity, a 10 mL aliquot of grape juice was diluted with 10 mL distilled water and titrated with 0.1 mol L^−1^ NaOH until reaching pH 8.1. The amount of NaOH used was converted to malic acid equivalents and expressed as g malic acid per 100 mL juice. This method was adapted from Dogan and Uyak (2020), with modification to reflect the dominant organic acid in grapes. Vitamin C content was measured using a Reflectoquant Plus 10 device (Merck RQflex Plus 10, Türkiye). A 0.5 mL juice sample was diluted to 5 mL with 0.5% oxalic acid. Merck ascorbic acid test strips (Cat. No. 116981) were dipped for 2 s, oxidized for 8 s, and read within 15 s. Results were expressed as mg ascorbic acid per 100 g fresh weight. The method was applied according to Ozturk et al. (2019), as cited in Dogan and Uyak (2020).

### 2.4 Extraction Procedure

Fresh grape berries were manually separated from clusters, washed with distilled water, and air‐dried at room temperature. For each genotype, berry skins and pulp tissues were homogenized together using a laboratory blender under cold conditions. Approximately 10 g of homogenized grape sample was extracted with 40 mL of methanol:water:formic acid solution (70:29:1, v/v/v). The extraction mixture was continuously shaken at 4°C for 24 h under dark conditions to minimize phenolic degradation. After extraction, samples were centrifuged at 10,000 rpm for 15 min at 4°C, and the supernatants were collected. The extracts were filtered through Whatman No. 1 filter paper and subsequently passed through a 0.45 μm membrane filter before biochemical and HPLC analyses. All extracts were prepared in triplicate and stored at −20°C until analysis.

### 2.5 Total Phenolic Content, Total Flavonoid Content and Total Monomeric Anthocyanin

Total phenolics were determined using the Folin–Ciocalteu method from methanolic grape extracts prepared as described above. Briefly, 600 μL of grape extract was mixed with 4.0 mL distilled water, followed by 100 μL Folin–Ciocalteu reagent and 300 μL of 2% sodium carbonate. After 2 h of incubation at room temperature, absorbance was read at 760 nm using a UV–Vis spectrophotometer (Shimadzu, Japan). Results were expressed as mg gallic acid equivalents (GAE) per 100 g fresh weight. Method adapted from Ozturk et al. (2019).

Flavonoid content was measured using a modified version of the method by Zhishen et al. (1999). A 600 μL aliquot of the methanolic grape extract was mixed with 3.7 mL methanol, followed by 100 μL of 10% aluminum nitrate and 0.1 M ammonium acetate, reaching a final volume of 4.5 mL. After 40 min of incubation in darkness, absorbance was read at 415 nm. Results were expressed as mg quercetin equivalents (QE) per 100 g fresh weight.

Anthocyanin content was determined using the pH differential method described by Giusti and Wrolstad (2003). Methanolic grape extracts were diluted separately in pH 1.0 and pH 4.5 buffer systems. Absorbance was measured at 533 and 700 nm. Results were calculated as cyanidin‐3‐glucoside equivalents and expressed as μg per g fresh weight.

### 2.6 Antioxidant Activity

#### 2.6.1 DPPH Radical Scavenging Activity

DPPH activity was measured using the method of Blois (1958). A 500 μL aliquot of methanolic grape extract was mixed with 2.5 mL ethanol, and 0.1 mM DPPH solution was added to reach a final volume of 4 mL. After vortexing and 30 min of incubation in darkness, absorbance was read at 517 nm. Results were expressed as mmol Trolox equivalents (TE) per 100 g fresh weight.

#### 2.6.2 Ferric Reducing Antioxidant Power (FRAP)

FRAP activity was measured following the protocol of Ozturk et al. (2019). A 100 μL aliquot of methanolic grape extract was mixed with 1.15 mL phosphate buffer (0.2 M, pH 6.7) and 1.25 mL potassium ferricyanide (1%). After 20 min at 50°C, the mixture was cooled and combined with 1.25 mL trichloroacetic acid (10%) and 0.25 mL ferric chloride (0.1%). Absorbance was read at 700 nm and expressed as mmol TE per 100 g fresh weight.

#### 2.6.3 Individual Phenolic Compounds

Individual phenolic compounds were quantified using high‐performance liquid chromatography (HPLC) according to the method of Rodríguez‐Delgado et al. (2001) with minor modifications. Prior to analysis, methanolic grape extracts prepared as described above were filtered through a 0.45 μm PVDF syringe filter (Millipore, USA). HPLC analyses were performed using an Agilent 1100 series HPLC system equipped with a diode array detector (DAD), quaternary pump, autosampler, and column oven. Separation was achieved using a reversed‐phase ODS C18 column (250 × 4.6 mm, 4 μm particle size, HiChrom, UK) maintained at 30°C. The mobile phase consisted of solvent A (water: acetic acid:methanol, 88:2:10, v/v/v) and solvent B (water: acetic acid: methanol, 8:2:90, v/v/v). A gradient elution program was applied as follows: 0–10 min, 100% A; 10–25 min, 85% A; 25–40 min, 70% A; 40–50 min, 50% A; 50–60 min, 100% B. The flow rate was maintained at 1.0 mL min^−1^ and injection volume was 20 μL. Phenolic compounds were identified by comparing their retention times and UV–Vis spectra with those of authentic external standards, including gallic acid, catechin, epicatechin, caffeic acid, chlorogenic acid, p‐coumaric acid, rutin, protocatechuic acid, p‐hydroxybenzoic acid, and aminobenzoic acid (Sigma‐Aldrich, USA). Quantification was performed using external calibration curves prepared at different concentration levels (R^2^ > 0.999). Detection wavelengths were set at 254, 280, and 320 nm depending on compound characteristics. Method validation parameters including linearity, repeatability, and sensitivity were evaluated prior to analysis. Limits of detection (LOD) and limits of quantification (LOQ) were determined based on signal‐to‐noise ratios of 3 and 10, respectively. All analyses were conducted in triplicate, and results were expressed as mg per 100 g fresh weight.

### 2.7 Statistical Analysis

Data normality was tested using the Kolmogorov Smirnov test, and variance homogeneity was assessed with Levene's test. One‐way ANOVA was performed using SAS 9.1, and significant differences among autochthonous genotypes were determined by Tukey's test at *p* < 0.05. Heatmap clustering and principal component analysis (PCA) were conducted using JMP Pro 17.

## 3 Results and Discussion

### 3.1 Cluster Properties

The cluster morphology of the 18 autochthonous grape genotypes exhibited substantial variation. Cluster length ranged from 9.00 cm (Keji Beyazı) to 18.16 cm (Dana Gözü 1), while cluster weight varied between 71.33 g (Kuru Üzüm) and 580.40 g (Sulu Üzüm). Cluster compactness was classified as “dense,” “moderate,” or “loose” by three trained observers according to OIV descriptors, ensuring standardized evaluation across genotypes. The compact cluster structure observed in Kaşper may increase susceptibility to fungal infections due to reduced air circulation within the cluster architecture. In contrast, Vakkas showed the loosest cluster morphology, with relatively low weight and width (Table 1). These findings are consistent with previous reports on Turkish highland viticulture, where cluster compactness and weight are strongly influenced by genotype and elevation‐related stress factors (Cakir et al. 2021; Polat et al. 2024). Similar variability was reported in plateau‐grown cultivars such as ‘Tilki Kuyruğu’ and ‘Göde Büzgülü’, which displayed distinct cluster morphologies under stress‐prone conditions. Indeed, the previous studies on local grape varieties grown in Anatolia reveal that traits such as cluster length, weight, and firmness vary depending on genotype differences (Celik 2006). The environmental stress factors (drought, low temperature, and soil structure) have been reported to play a decisive role in cluster compactness, particularly in varieties grown in high‐altitude regions (Eyduran et al. 2015). In addition, the studies conducted in Van and its surrounding areas show that local varieties have a wide morphological variation and that this diversity constitutes an important genetic resource for viticulture (Bas 2018). However, cluster density is not only a morphological factor but also related to quality. Previous studies have indicated that varieties with compact clusters may be more susceptible to fungal and fungal diseases, while varieties with loose clusters are more prone to disease (Pehlivan and Uzun 2015). Therefore, it is emphasized that cluster morphology should be evaluated in terms of both yield and quality criteria.

**TABLE 1:** Cluster morphology parameters of 18 autochthonous grape genotypes.

Variety	Cluster length (cm)	Cluster width (cm)	Cluster weight (g)	Compactness
Beyaz Üzüm	13.75 ± 3.10 b	8.25 ± 0.96 c	442.00 ± 89.32 b	Moderate
Dana Gözü 1	18.16 ± 1.91 a	13.00 ± 2.38 a	302.50 ± 60.18 c	Moderate
Dana Gözü 2	16.25 ± 1.53 a	10.75 ± 1.15 b	297.20 ± 57.40 c	Moderate
Dımışkı	13.00 ± 2.48 b	8.25 ± 1.50 c	559.20 ± 87.21 a	Moderate
Elazığ Kırmızısı	11.00 ± 2.59 c	7.25 ± 2.19 d	206.10 ± 50.79 d	Moderate
Güz Beyazı	13.60 ± 2.59 b	7.00 ± 1.68 d	155.62 ± 29.07 e	Moderate
Kaşper	10.06 ± 1.52 d	8.60 ± 1.14 c	276.50 ± 85.32 c	Very Dense
Keçi Memesi	17.60 ± 1.52 a	8.40 ± 1.14 c	167.30 ± 26.39 e	Moderate
Keji Beyazı	9.00 ± 1.44 d	7.21 ± 1.39 d	286.30 ± 79.12 c	Dense
Kırmızı Üzüm	14.98 ± 1.42 b	13.59 ± 0.94 a	236.60 ± 87.36 d	Dense
Kuru Üzüm	11.89 ± 0.82 c	11.23 ± 0.76 b	71.33 ± 22.66 f	Dense
Lüle Beyazı	12.33 ± 1.53 c	8.30 ± 0.58 c	270.10 ± 38.59 c	Moderate
Sinciri 1	10.17 ± 1.72 d	6.50 ± 0.84 e	175.17 ± 15.73 e	Dense
Sinciri 2	13.40 ± 2.50 b	5.95 ± 0.93 e	165.16 ± 32.30 e	Moderate
Sulu Üzüm	13.25 ± 2.22 b	7.75 ± 1.26 d	580.40 ± 207.55 a	Dense
Vakkas	13.28 ± 1.89 b	6.40 ± 0.79 e	142.40 ± 40.28 e	Loose
Vakkas Dişi	15.00 ± 1.63 a	9.00 ± 2.16 c	570.00 ± 99.90 a	Moderate
Vakkas Erkek	15.60 ± 2.41 a	10.20 ± 0.84 b	247.00 ± 49.78 d	Dense

*Note:* Means in columns with the same letter do not differ according to Tukey's test at *p* < 0.05.

### 3.2 Berry Properties

Berry length ranged from 11.89 mm (Kuru Üzüm) to 22.95 mm (Elazığ Kırmızısı), while berry weight varied between 1.04 g and 4.64 g (Dana Gözü 1). Most genotypes exhibited either Broad Oval or Short Oval berry shapes, with minor differences in elongation and width ratios. These shape classifications were based on OIV descriptors and confirmed by calibrated measurements. Larger berry size in genotypes such as Lüle Beyazı and Vakkas Dişi may contribute positively to market preference and fresh consumption potential (Table 2). These findings support previous pomological evaluations of Turkish table grape cultivars, where berry shape and size—particularly oval forms with balanced dimensions—are key determinants of consumer appeal and processing suitability (Cangi and Altun 2015). The ampelographic studies on grape varieties grown in different regions of Türkiye have reported significant variation in berry size, weight, and shape, with larger, oval berries being more popular in the market, particularly for table grapes (Celik 2006). The studies on local cultivars in Van and Eastern Anatolia indicate that larger‐grained varieties are preferred for table grapes, while smaller, denser‐skinned varieties are often used for drying or wine production (Bas 2018). It has also been reported that coarse‐grained varieties generally contain a medium amount of seeds, a trait that increases consumer appreciation (Uyak et al. 2020). This supports the advantage of genotypes such as Lüle Beyazı and Vakkas Dişi for table consumption.

**TABLE 2:** Berry traits of autochthonous grape genotypes.

Variety	Berry length (mm)	Berry width (mm)	Berry weight (g)	Shape
Beyaz Üzüm	17.71 ± 1.19 b	16.74 ± 1.37 b	3.29 ± 0.68 b	Broad Oval
Dana Gözü 1	20.24 ± 1.33 a	18.47 ± 1.89 a	4.64 ± 1.04 a	Short Oval
Dana Gözü 2	17.19 ± 1.19 b	15.57 ± 1.11 c	2.74 ± 0.59 c	Broad Oval
Dımışkı	13.73 ± 1.38 e	12.93 ± 1.05 e	1.67 ± 0.60 e	Broad Oval
Elazığ Kırmızısı	22.95 ± 1.25 a	22.84 ± 0.83 a	3.16 ± 0.52 b	Broad Oval
Güz Beyazı	15.30 ± 0.88 d	16.69 ± 0.68 b	2.17 ± 0.34 d	Broad Oval
Kaşper	16.53 ± 0.88 c	14.07 ± 0.68 d	2.17 ± 0.42 d	Short Oval
Keçi Memesi	18.18 ± 1.22 b	15.37 ± 0.87 c	2.98 ± 0.56 c	Short Oval
Keji Beyazı	16.95 ± 1.22 b	15.36 ± 0.87 c	2.49 ± 0.49 c	Short Oval
Kırmızı Üzüm	14.98 ± 1.42 d	13.59 ± 0.94 d	1.81 ± 0.28 e	Broad Oval
Kuru Üzüm	11.89 ± 0.82 f	11.23 ± 0.76 f	1.04 ± 0.19 f	Broad Oval
Lüle Beyazı	20.76 ± 1.82 a	18.17 ± 1.48 a	4.34 ± 1.21 a	Short Oval
Sinciri 1	14.04 ± 1.10 d	14.16 ± 0.92 d	1.84 ± 0.31 e	Broad Oval
Sinciri 2	15.59 ± 0.93 d	15.02 ± 1.15 c	2.12 ± 0.31 d	Broad Oval
Sulu Üzüm	15.71 ± 1.18 d	15.23 ± 1.92 c	2.53 ± 0.66 c	Broad Oval
Vakkas	15.44 ± 0.88 d	13.13 ± 0.81 d	1.89 ± 0.27 e	Short Oval
Vakkas Dişi	15.63 ± 0.87 d	14.16 ± 0.81 d	2.03 ± 0.23 d	Broad Oval
Vakkas Erkek	15.71 ± 0.69 d	14.32 ± 0.71 d	2.02 ± 0.29 d	Broad Oval

*Note:* Means in columns with the same letter do not differ according to Tukey's test at *p* < 0.05.

### 3.3 Skin and Seed Properties

Skin thickness and seed characteristics varied significantly among the 18 autochthonous grape genotypes. Skin thickness ranged from 0.21 mm (Dana Gözü 1) to 0.56 mm (Kaşper), with Kaşper, Keçi Memesi, and Vakkas genotypes exhibiting the thickest skins (Table 3). These variations may influence postharvest durability and suitability for drying, as thicker skins are generally associated with enhanced resistance to mechanical damage and water loss (Costa et al. 2014). Skin thickness was predominantly classified as “thin” or “medium” by three trained observers using standardized OIV descriptors, ensuring consistency and reproducibility. Skin color variation among genotypes was generally consistent with expected differences in anthocyanin pigmentation. Genotypes were grouped into three main color categories: green‐yellow (Beyaz Üzüm, Sinciri 1), red‐black (Dımışkı, Vakkas Dişi), and dark red‐purple (Dana Gözü 1, Kaşper, Kırmızı Üzüm) (Table 3).

**TABLE 3:** Skin thickness and seed traits of autochthonous grape genotypes.

Variety	Skin thickness (mm)	Seed number	Seed weight (mg/seed)	Skin color
Beyaz Üzüm	0.32 ± 0.04 f	2.78 ± 0.21 c	26.21 ± 4.32 g	Green‐Yellow
Dana Gözü 1	0.21 ± 0.03 g	2.44 ± 0.18 d	36.17 ± 3.82 d	Dark Red‐Purple
Dana Gözü 2	0.32 ± 0.05 f	2.35 ± 0.17 d	38.18 ± 3.85 c	Dark Red‐Purple
Dımışkı	0.45 ± 0.06 e	3.02 ± 0.22 b	27.33 ± 4.12 g	Red‐Black
Elazığ Kırmızısı	0.32 ± 0.04 f	3.15 ± 0.24 b	36.57 ± 3.61 d	Red
Güz Beyazı	0.45 ± 0.05 e	3.05 ± 0.23 b	32.51 ± 3.96 e	Green‐Yellow
Kaşper	0.56 ± 0.07 c	3.28 ± 0.26 a	46.57 ± 6.08 a	Dark Red‐Purple
Keçi Memesi	0.52 ± 0.06 d	3.10 ± 0.22 b	39.62 ± 3.82 c	Dark Red‐Purple
Keji Beyazı	0.32 ± 0.04 f	2.89 ± 0.20 c	36.82 ± 4.45 d	Green‐Yellow
Kırmızı Üzüm	0.32 ± 0.04 f	3.35 ± 0.27 a	41.66 ± 4.31 b	Dark Red‐Purple
Kuru Üzüm	0.32 ± 0.04 f	2.98 ± 0.21 b	40.16 ± 4.59 b	Dark Red‐Purple
Lüle Beyazı	0.32 ± 0.04 f	3.12 ± 0.23 b	45.52 ± 4.91 a	Yellow‐Green
Sinciri 1	0.32 ± 0.04 f	3.24 ± 0.25 a	44.05 ± 0.62 a	Green‐Yellow
Sinciri 2	0.32 ± 0.04 f	3.38 ± 0.28 a	44.24 ± 0.22 a	Green‐Yellow
Sulu Üzüm	0.32 ± 0.04 f	3.12 ± 0.23 b	35.75 ± 4.45 d	Dark Red‐Purple
Vakkas	0.45 ± 0.05 e	3.15 ± 0.24 b	39.12 ± 4.05 c	Dark Red‐Purple
Vakkas Dişi	0.45 ± 0.05 e	3.26 ± 0.26 a	40.13 ± 4.82 b	Red‐Black
Vakkas Erkek	0.45 ± 0.06 e	2.19 ± 0.16 d	38.95 ± 5.03 c	Dark Red‐Purple

*Note:* Means in columns with the same letter do not differ according to Tukey's test at *p* < 0.05.

Seed number per berry ranged from 2.19 (Vakkas Erkek) to 3.38 (Sinciri 2), while seed weight varied between 26.21 mg (Beyaz Üzüm) and 46.57 mg (Kaşper). Genotypes such as Lüle Beyazı, Sinciri 2, and Kaşper had heavier seeds, which may affect consumer preference and processing efficiency. In contrast, genotypes with lower seed load and thinner skins such as Beyaz Üzüm and Keji Beyazı may be more suitable for fresh consumption due to their softer texture and reduced seed burden (Table 3). These traits are critical for evaluating grape suitability for different uses. Thicker skins and higher seed mass may favor drying and juice extraction, while thinner skins and fewer seeds are preferred for table grapes. The observed variation also supports the hypothesis that genotype and microclimate jointly influence seed development and skin pigmentation. Previous studies have also shown that skin thickness has important effects on grape storage time, suitability for drying and resistance to fungal infections (Pehlivan and Uzun 2015). It has been reported that, especially in the local varieties grown in Eastern and Central Anatolia, large and thick‐shelled grains are preferred for drying, while thin‐shelled varieties are preferred for table use (Bas 2018).

As expected, darker‐skinned genotypes generally exhibited higher anthocyanin concentrations than light‐skinned cultivars (Baydar 2006; Eyduran et al. 2015). In addition, it has been reported that seed number and weight are decisive in consumer preferences, especially for table grapes, and varieties with fewer seeds and softer skins provide an advantage in terms of market value (Yilmaz and Toledo 2004; Bozan et al. 2008). In this context, it can be said that the variation observed in the study reflects the effect of the microclimatic conditions in which it is grown along with the genotype differences, and therefore should be taken into consideration both in genetic breeding programs and regional production strategies (Eyduran et al. 2014; Uyak et al. 2020).

### 3.4 Chemical Composition

Must yield among the 18 autochthonous grape genotypes ranged from 13.50 mL/100 g (Kuru Üzüm) to 19.20 mL/100 g (Dana Gözü 1), with the highest values observed in Sulu Üzüm, Vakkas Erkek, and Beyaz Üzüm (Table 4). These differences reflect genotype‐dependent variation in berry flesh‐to‐skin ratio and juice retention capacity, which are critical parameters for both table and processing grapes (Costa et al. 2014; Balbaba and Bagci 2022).

**TABLE 4:** Must yield, soluble solids content (SSC), titratable acidity, and vitamin C content of autochthonous grape genotypes.

Variety	Must yield (mL/100 g)	SSC (%)	Titratable acidity (%)	Vitamin C (mg/100 g)
Beyaz Üzüm	18.00 ± 0.25 ab	19.26 ± 0.55 bcd	1.03 ± 0.02 ab	15.63 ± 0.09 d
Dana Gözü 1	19.20 ± 0.52 a	16.43 ± 1.10 ef	1.04 ± 0.03 ab	3.83 ± 0.03 k
Dana Gözü 2	17.35 ± 1.18 ab	17.83 ± 0.15 de	1.02 ± 0.04 ab	4.50 ± 0.23 k
Dımışkı	16.55 ± 1.35 bc	23.33 ± 0.60 a	1.02 ± 0.06 ab	23.27 ± 0.15 a
Elazığ Kırmızısı	16.50 ± 0.50 bc	16.90 ± 1.73 def	1.14 ± 0.08 ab	15.70 ± 0.17 cd
Güz Beyazı	17.25 ± 0.42 b	15.90 ± 1.33 f	1.03 ± 0.88 ab	8.53 ± 0.15 h
Kaşper	15.50 ± 0.68 cd	15.90 ± 0.80 f	1.03 ± 0.04 ab	9.03 ± 0.20 h
Keçi Memesi	14.50 ± 1.65 de	16.26 ± 0.59 f	1.05 ± 0.05 ab	6.47 ± 0.20 j
Keji Beyazı	14.25 ± 1.38 ef	16.83 ± 1.12 ef	0.82 ± 0.10 b	9.00 ± 0.06 h
Kırmızı Üzüm	17.25 ± 0.75 b	16.70 ± 0.06 ef	1.02 ± 0.02 ab	6.13 ± 0.15 j
Kuru Üzüm	13.50 ± 0.59 f	19.40 ± 0.25 bc	1.18 ± 0.01 ab	13.67 ± 0.26 e
Lüle Beyazı	16.50 ± 0.57 bc	18.80 ± 0.31 cd	0.87 ± 0.05 b	11.13 ± 0.15 g
Sinciri 1	17.00 ± 0.50 b	21.18 ± 0.15 b	0.97 ± 0.02 b	6.53 ± 0.20 j
Sinciri 2	17.00 ± 0.50 b	20.67 ± 2.21 b	0.82 ± 0.10 b	7.40 ± 1.05 i
Sulu Üzüm	15.50 ± 0.60 cd	17.80 ± 1.10 de	1.39 ± 0.14 a	20.13 ± 0.20 b
Vakkas	14.50 ± 0.40 e	22.50 ± 0.40 a	1.02 ± 0.09 ab	16.53 ± 0.03 c
Vakkas Dişi	15.50 ± 0.75 cd	20.53 ± 0.50 b	0.95 ± 0.09 b	11.23 ± 0.15 g
Vakkas Erkek	17.50 ± 1.15 ab	18.10 ± 0.64 de	0.91 ± 0.01 b	12.80 ± 0.17 f

*Note:* Means in columns with the same letter do not differ according to Tukey's test at *p* < 0.05.

The variability observed in SSC and titratable acidity indicates important genotype‐specific differences in ripening behavior and sugar–acid balance. SSC varied, ranging from 15.90% (Kaşper, Güz Beyazı) to 23.33% (Dımışkı). Genotypes such as Kuru Üzüm, Vakkas, and Vakkas Dişi also exhibited elevated SSC levels, suggesting higher sugar accumulation (Table 4). These results are consistent with previous studies reporting SSC values between 14% and 24% in Turkish cultivars grown under highland conditions (Ozturk et al. 2019; Cakir et al. 2021). Titratable acidity, expressed as malic acid equivalent, ranged from 0.82% (Keji Beyazı, Sinciri 2) to 1.39% (Sulu Üzüm), indicating a broad spectrum of sourness perception across genotypes. While most varieties clustered around 1.00%, the elevated TA in Sulu Üzüm may contribute to its balanced flavor profile, especially when paired with high SSC. The variation in ascorbic acid content suggests that antioxidant metabolism differs substantially among local grape genotypes. Genotypes such as Sulu Üzüm, Vakkas, and Elazığ Kırmızısı also exhibited high ascorbic acid levels, suggesting potential for antioxidant‐rich fresh consumption (Table 4). These values are in agreement with previous reports on Turkish grape cultivars, where vitamin C content was shown to vary widely depending on genotype and ripening stage (Ozturk et al. 2019; Kaya et al. 2025). Overall, genotypes like Dımışkı, Sulu Üzüm, and Vakkas Dişi stand out for their favorable combination of high SSC, moderate acidity, and elevated vitamin C, making them promising candidates for fresh consumption and further nutritional evaluation. Similarly, the studies on grapes grown in the Elazığ and Malatya regions have revealed that SSC, organic acids, and vitamin C content vary significantly depending on genotype and ecological conditions (Duran 2014). Specifically, high‐SSC varieties are reported to be suitable for drying and winemaking, while high‐ascorbic acid varieties are reported to be preferred for fresh consumption and functional food potential (Eyduran et al. 2014). Additionally, the studies conducted in Eastern Anatolia indicate that grape juice yield and must composition in local grape genotypes vary depending on environmental conditions, and that these varieties offer an important genetic resource in terms of biochemical traits (R. I. Gazioglu Sensoy 2015; Bas 2018). These differences are also closely related to vineyard management, soil properties, and harvest timing (Barroso et al. 2017). Therefore, the findings obtained in this study confirm that local varieties should be evaluated in terms of must yield and chemical composition as well as their morphological differences, and that genotypes containing high SSC and vitamin C in particular may provide useful biochemical characteristics for future grape improvement and utilization studies (Karadogan and Keskin 2017; Fidan et al. 2018).

### 3.5 Total Phenolic, Flavonoid and Anthocyanin Content

The total phenolic content (TPC) of the 18 autochthonous grape genotypes ranged from 52.70 mg GAE/100 g (Beyaz Üzüm) to 249.73 mg GAE/100 g (Dana Gözü 2), indicating substantial biochemical diversity (Table 5). Genotypes such as Sulu Üzüm, Vakkas Dişi, and Dımışkı also exhibited elevated phenolic levels, consistent with previous findings that darker‐skinned grapes tend to accumulate higher concentrations of phenolic compounds (Costa et al. 2014).

**TABLE 5:** Total phenolic, flavonoid, anthocyanin contents, and antioxidant activity of autochthonous grape genotypes.

Variety	Total phenolic (mg GAE/100 g)	Total flavonoid (mg QE/100 g)	Total anthocyanin (μg cy‐3‐glu/g)	DPPH (mmol TE/100 g)	FRAP (mmol TE/100 g)
Beyaz Üzüm	52.70 ± 1.67 j	42.80 ± 2.95 k	—	4.25 ± 0.09 hi	4.68 ± 0.18 g
Dana Gözü 1	84.07 ± 1.52 f–i	67.57 ± 3.75 ef	8.34 ± 0.15 a	5.69 ± 0.17 f	5.46 ± 0.14 f
Dana Gözü 2	249.73 ± 24.29 a	53.80 ± 3.95 hi	2.21 ± 0.11 e	3.45 ± 0.20 jk	4.28 ± 0.08 h
Dımışkı	127.43 ± 3.03 cd	91.53 ± 4.62 a	—	18.45 ± 0.81 a	7.37 ± 0.10 c
Elazığ Kırmızısı	87.43 ± 1.68 f‐h	69.37 ± 3.05 d‐f	5.66 ± 0.13 d	8.74 ± 0.12 e	7.36 ± 0.21 c
Güz Beyazı	63.37 ± 1.96 ij	43.70 ± 5.10 k	—	4.68 ± 0.23 h	6.15 ± 0.09 e
Kaşper	76.33 ± 0.32 hi	60.57 ± 4.85 gh	—	10.97 ± 0.06 d	4.18 ± 0.08 hi
Keçi Memesi	72.53 ± 3.44 hi	59.40 ± 3.50 g‐i	8.17 ± 0.04 a	4.59 ± 0.12 hi	5.71 ± 0.19 f
Keji Beyazı	99.70 ± 2.83 ef	53.47 ± 3.46 ij	—	6.05 ± 0.09 f	6.46 ± 0.24 de
Kırmızı Üzüm	104.00 ± 4.27 de	60.57 ± 0.85 gh	8.03 ± 0.04 a	3.89 ± 0.11 ij	3.81 ± 0.12 ij
Kuru Üzüm	97.53 ± 2.75 fg	46.87 ± 4.50 jk	—	13.17 ± 0.31 c	10.47 ± 0.08 b
Lüle Beyazı	87.43 ± 2.37 f‐h	54.53 ± 3.35 hi	—	3.09 ± 0.07 k	3.56 ± 0.08 j
Sinciri 1	119.10 ± 3.02 de	63.47 ± 3.65 fg	—	10.79 ± 0.11 d	6.57 ± 0.19 d
Sinciri 2	76.37 ± 4.39 hi	46.60 ± 3.60 k	—	2.96 ± 0.05 k	5.56 ± 0.16 f
Sulu Üzüm	163.07 ± 3.79 b	81.10 ± 6.55 bc	7.52 ± 0.20 b	15.12 ± 0.12 b	14.16 ± 0.12 a
Vakkas	102.40 ± 1.92 de	87.40 ± 3.86 ab	—	5.44 ± 0.06 fg	7.60 ± 0.06 c
Vakkas Dişi	143.67 ± 4.77 c	76.10 ± 2.72 cd	7.00 ± 0.06 c	1.98 ± 0.02 L	2.37 ± 0.02 k
Vakkas Erkek	77.27 ± 2.97 g‐i	71.23 ± 6.45 de	—	4.85 ± 0.10 gh	5.74 ± 0.08 f

*Note:* Means in columns with the same letter do not differ according to Tukey's test at *p* < 0.05.

Total flavonoid content (TFC) varied between 42.80 mg QE/100 g (Beyaz Üzüm) and 91.53 mg QE/100 g (Dımışkı). High flavonoid levels were also observed in Vakkas, Sulu Üzüm, and Elazığ Kırmızısı, supporting their antioxidant potential and pigmentation intensity (Table 5). These compounds are known to contribute significantly to radical scavenging activity and are typically enriched in red and black grape varieties (Xia et al. 2010).

Anthocyanin content was detected in nine genotypes, ranging from 2.21 μg cy‐3‐glu/g (Dana Gözü 2) to 8.34 μg cy‐3‐glu/g (Dana Gözü 1) (Table 5). Genotypes with dark‐colored berry skins generally exhibited higher anthocyanin concentrations, which was consistent with expected pigmentation patterns (Zhao et al. 2024). Anthocyanins were not detected in white‐skinned genotypes, consistent with their lack of red‐purple pigmentation (Rodríguez‐López et al. 2022). Overall, genotypes like Dımışkı, Sulu Üzüm, and Vakkas Dişi stand out for their combined richness in phenolics, flavonoids, and anthocyanins. These profiles indicate substantial biochemical diversity and antioxidant potential among the evaluated genotypes and may warrant further biological and nutritional investigations. Indeed, the studies conducted in Turkey have revealed significant variability in phenolic compound, flavonoid, and anthocyanin contents among grape varieties. Although phenolic compounds are widely associated with antioxidant‐related properties, their biological effects may vary depending on bioavailability, metabolism, and digestion. High phenolic content has been reported in genotypes originating from Eastern Anatolia, with these levels being particularly pronounced in darker‐colored varieties (Eyduran et al. 2014; Uyak et al. 2020). Similarly, studies by Baydar (2006) emphasize the significant contribution of phenolic compounds to the antioxidant capacity of grapes and wine quality. Furthermore, analyses by Vural (2011) and R. Gazioglu Sensoy (2012). indicate that anthocyanin and phenolic contents are critical for both fresh consumption and industrial use. High anthocyanin accumulation in dark‐colored varieties may contribute to antioxidant‐related properties and biochemical richness (Karadogan and Keskin 2017). Previous studies have also indicated that flavonoids are particularly abundant in red and black grapes, and that these compounds contribute to both color formation and free radical scavenging capacity (Kafkas et al. 2006). These findings demonstrate that the evaluated genotypes possess considerable phenolic and antioxidant diversity that may warrant further biological and nutritional investigations.

### 3.6 Antioxidant Activity (DPPH and FRAP)

The antioxidant capacity of the 18 autochthonous grape genotypes, evaluated through DPPH and FRAP assays, revealed substantial variation. DPPH radical scavenging activity ranged from 1.98 mmol TE/100 g (Vakkas Dişi) to 18.45 mmol TE/100 g (Dımışkı), while FRAP values varied between 2.37 mmol TE/100 g (Vakkas Dişi) and 14.16 mmol TE/100 g (Sulu Üzüm) (Table 5). These results confirm that antioxidant potential is strongly genotype‐dependent and closely linked to phenolic and flavonoid content (Xia et al. 2010; Costa et al. 2014).

Genotypes such as Dımışkı, Sulu Üzüm, and Kuru Üzüm may represent promising candidates for future studies investigating grape antioxidant composition (Table 5). This supports previous findings that phenolic compounds—particularly flavonoids and anthocyanins—play a central role in neutralizing free radicals and reducing oxidative stress (Zhao et al. 2024).

Interestingly, Kaşper and Sinciri 1, despite having moderate phenolic content, showed relatively high DPPH activity. This may indicate the presence of specific radical‐scavenging compounds or synergistic interactions among minor phenolics, organic acids, or vitamin C. In contrast, Vakkas Dişi, while rich in anthocyanins and flavonoids, exhibited the lowest antioxidant activity, indicating that pigment concentration alone may not fully predict antioxidant performance (Table 5). These findings emphasize the importance of evaluating multiple antioxidant parameters simultaneously rather than relying on single markers. Genotypes such as Dımışkı, Sulu Üzüm, and Kuru Üzüm may represent promising genotypes for future studies investigating the biological relevance of grape antioxidant compounds due to their consistent antioxidant profiles across both assays. Similarly, studies conducted in Türkiye reported strong correlations between phenolic compounds and ascorbic acid content and antioxidant capacity (Baydar 2006). High DPPH and FRAP activities in grape varieties originating from Eastern and Southeastern Anatolia have been reported to be associated with anthocyanin and flavonoid accumulation (Eyduran et al. 2014). Vural (2011) and R. Gazioglu Sensoy (2012) emphasize that antioxidant capacity in native grape varieties varies significantly among varieties, and that these differences are influenced by environmental factors as well as phenolic profiles. Furthermore, analyses on Karaerik clones by Karadogan and Keskin (2017) confirmed the relationship between high phenolic content and strong antioxidant activity. These results demonstrate the biochemical richness and antioxidant‐related properties of varieties such as Dımışkı, Sulu Üzüm, and Kuru Üzüm; however, the physiological relevance of these compounds requires further biological validation. Furthermore, since pigment density alone is not a sufficient indicator, it once again demonstrates the need to use multiple parameters in antioxidant capacity assessments (R. Gazioglu Sensoy 2012). However, it should be emphasized that DPPH and FRAP assays are in vitro chemical methods and do not directly reflect in vivo antioxidant effects, bioavailability, digestion, or biological activity in humans. Therefore, the antioxidant potential observed in this study should be interpreted cautiously until supported by further cellular, physiological, and clinical investigations.

### 3.7 Individual Phenolic Compounds

High‐performance liquid chromatography (HPLC) analysis revealed significant variation in the concentration of individual phenolic compounds among the 18 autochthonous grape genotypes (Tables 6 and 7). The most abundant compounds across genotypes were catechin, epicatechin, p‐hydroxybenzoic acid, and aminobenzoic acid, with several cultivars exhibiting distinctive biochemical profiles. In addition to flavonoids and hydroxycinnamic acids (Table 6), benzoic acid derivatives and other minor phenolics were quantified and are presented in Table 7. Catechin levels ranged from 0.00 mg/100 g (Dana Gözü 2) to 252.35 mg/100 g (Sulu Üzüm), with Dımışkı, Kuru Üzüm, and Dana Gözü 1 also exhibiting elevated concentrations. Catechin is a key flavanol with potent antioxidant properties, and its accumulation is often associated with berry skin thickness and genotype‐specific biosynthetic capacity (Costa et al. 2014). Epicatechin was highest in Dımışkı (30.30 mg/100 g) and Sulu Üzüm (26.56 mg/100 g), while several genotypes such as Dana Gözü 2 and Kırmızı Üzüm showed minimal levels. This compound contributes to radical scavenging and has been linked to cardiovascular protective effects (Rodríguez‐López et al. 2022). Rutin, a glycosylated flavonoid, was most abundant in Sulu Üzüm (4.36 mg/100 g) and Sinciri 1 (2.93 mg/100 g), while white‐skinned genotypes such as Dana Gözü 2 and Kırmızı Üzüm showed no detectable levels. Rutin is known for its anti‐inflammatory and vasoprotective properties and is often concentrated in pigmented cultivars (Zhao et al. 2024). p‐Coumaric acid and caffeic acid were highest in Sulu Üzüm and Dımışkı, with values of 5.95 mg/100 g and 3.96 mg/100 g, respectively. These hydroxycinnamic acids are precursors in the phenylpropanoid pathway and contribute to both antioxidant activity and color stability (Moradi et al. 2024).

**TABLE 6:** Individual phenolic compounds in local grape genotypes.

Variety	Catechin (mg/100 g)	Epicatechin	Rutin	p‐coumaric acid	Caffeic acid
Beyaz Üzüm	20.22 ± 1.82 hi	5.74 ± 0.06 f	3.12 ± 0.06 b	0.00 ± 0.00 f	0.21 ± 0.06 d–f
Dana Gözü 1	71.17 ± 1.82 d	4.46 ± 0.06 hi	1.37 ± 0.06 cde	0.00 ± 0.00 f	0.20 ± 0.06 d–f
Dana Gözü 2	0.00 ± 0.00 m	0.00 ± 0.00 m	0.00 ± 0.00 i	0.00 ± 0.00 f	0.00 ± 0.00 h
Dımışkı	156.93 ± 2.44 b	30.30 ± 0.14 a	2.02 ± 0.14 c	2.88 ± 0.14 b	3.96 ± 0.14 a
Elazığ Kırmızısı	12.87 ± 1.82 jk	2.33 ± 0.06 k	0.87 ± 0.06 e–h	0.02 ± 0.01 f	0.10 ± 0.01 gh
Güz Beyazı	25.75 ± 1.82 gh	6.94 ± 0.06 e	0.50 ± 0.06 f–i	1.45 ± 0.06 cd	0.00 ± 0.00 h
Kaşper	5.97 ± 1.82 k–m	2.71 ± 0.06 jk	0.81 ± 0.06 e–h	1.31 ± 0.06 cd	0.49 ± 0.06 c
Keçi Memesi	22.25 ± 1.82 hi	7.32 ± 0.06 de	0.35 ± 0.06 g–i	3.16 ± 0.06 b	0.36 ± 0.06 cd
Keji Beyazı	25.57 ± 2.97 gh	5.07 ± 0.06 gh	0.55 ± 0.06 f–i	0.00 ± 0.00 f	0.42 ± 0.06 c
Kırmızı Üzüm	4.40 ± 1.82 lm	1.09 ± 0.06 L	0.00 ± 0.00 i	0.00 ± 0.00 f	0.21 ± 0.06 d–f
Kuru Üzüm	85.80 ± 2.22 c	18.05 ± 0.06 c	0.98 ± 0.06 efg	1.93 ± 0.06 c	0.80 ± 0.06 b
Lüle Beyazı	52.79 ± 1.82 e	7.85 ± 0.06 d	1.70 ± 0.06 cd	0.00 ± 0.00 f	0.21 ± 0.06 d–f
Sinciri 1	30.67 ± 1.82 fg	7.68 ± 0.09 d	2.93 ± 0.09 b	0.41 ± 0.06 f	0.51 ± 0.09 c
Sinciri 2	9.93 ± 1.82 j–l	3.00 ± 0.12 j	0.21 ± 0.01 hi	1.17 ± 0.06 e	0.08 ± 0.01 gh
Sulu Üzüm	252.35 ± 3.55 a	26.56 ± 0.92 b	4.36 ± 0.92 a	5.95 ± 0.92 a	0.34 ± 0.06 c–e
Vakkas	25.71 ± 4.33 gh	5.19 ± 0.06 fg	0.84 ± 0.06 e–h	0.00 ± 0.00 f	0.12 ± 0.03 gh
Vakkas Dişi	36.14 ± 2.97 f	6.80 ± 0.06 e	1.20 ± 0.06 def	0.00 ± 0.00 f	0.08 ± 0.02 gh
Vakkas Erkek	16.09 ± 1.82 ij	4.02 ± 0.06 i	0.49 ± 0.06 f–i	0.00 ± 0.00 f	0.17 ± 0.01 f–h

*Note:* Means in columns with the same letter do not differ according to Tukey's test at *p* < 0.05.

**TABLE 7:** Individual phenolic compounds in local grape genotypes (continued).

Variety	Chlorogenic acid (mg/100 g)	Aminobenzoic acid	Protocatechuic acid	p‐hydroxybenzoic acid
Beyaz Üzüm	0.00 ± 0.00 g	0.77 ± 0.06 gh	0.00 ± 0.00 j	65.12 ± 1.82 i
Dana Gözü 1	0.00 ± 0.00g	2.02 ± 0.06 c–e	0.18 ± 0.01 ghi	38.04 ± 1.82 k
Dana Gözü 2	0.00 ± 0.00 g	0.00 ± 0.00 i	0.00 ± 0.00 j	0.00 ± 0.00 m
Dımışkı	11.46 ± 0.76 a	4.70 ± 0.14 b	0.47 ± 0.04 e	168.58 ± 2.44 c
Elazığ Kırmızısı	0.00 ± 0.00 g	0.25 ± 0.06 hi	0.40 ± 0.06 ef	19.66 ± 1.82 l
Güz Beyazı	2.10 ± 0.12 ef	0.38 ± 0.06 g–i	0.29 ± 0.06 fg	89.44 ± 1.82 h
Kaşper	1.48 ± 0.40 f	1.50 ± 0.06 ef	0.82 ± 0.06 c	68.02 ± 1.15 h
Keçi Memesi	0.00 ± 0.00 f	2.15 ± 0.06 c–e	0.21 ± 0.06 gh	99.64 ± 1.82 g
Keji Beyazı	0.00 ± 0.00 g	2.15 ± 0.06 c–e	0.13 ± 0.03 hi	156.40 ± 2.97 d
Kırmızı Üzüm	0.00 ± 0.00 g	0.45 ± 0.06 g–i	0.11 ± 0.01 h–j	66.48 ± 1.82 i
Kuru Üzüm	7.54 ± 0.30 b	2.44 ± 0.06 cd	1.37 ± 0.06 a	121.27 ± 2.22 e
Lüle Beyazı	0.00 ± 0.00 g	2.12 ± 0.06 c–e	0.00 ± 0.00 j	94.34 ± 1.82 gh
Sinciri 1	4.85 ± 0.58 d	1.61 ± 0.09 ef	0.60 ± 0.09 d	55.94 ± 2.88 j
Sinciri 2	0.00 ± 0.00 g	0.55 ± 0.06 g–i	0.00 ± 0.00 j	52.71 ± 1.82 j
Sulu Üzüm	6.03 ± 0.58 c	8.90 ± 0.92 a	1.14 ± 0.06 b	114.28 ± 3.55 f
Vakkas	0.00 ± 0.00 g	1.93 ± 0.06 de	0.07 ± 0.03 ij	265.41 ± 4.33 a
Vakkas Dişi	2.58 ± 0.29 e	2.73 ± 0.06 c	0.15 ± 0.03 hi	192.25 ± 2.97 b
Vakkas Erkek	1.51 ± 0.14 f	1.05 ± 0.06 fg	0.10 ± 0.01 h–j	90.08 ± 1.82 h

*Note:* Means in columns with the same letter do not differ according to Tukey's test at *p* < 0.05.

Among the benzoic acid derivatives, p‐hydroxybenzoic acid showed the widest range, from 0.00 mg/100 g (Dana Gözü 2) to 265.41 mg/100 g (Vakkas), indicating strong genotype‐specific accumulation. Genotypes such as Vakkas Dişi, Dımışkı, and Keji Beyazı also exhibited high levels, suggesting potential for antimicrobial and preservative applications (Balbaba and Bagci 2022). Chlorogenic acid, aminobenzoic acid, and protocatechuic acid were most concentrated in Dımışkı, Sulu Üzüm, and Kuru Üzüm, respectively. Chlorogenic acid is known for its role in oxidative stress modulation, while protocatechuic acid is a metabolite of anthocyanins with anti‐inflammatory activity. Aminobenzoic acid, found in high levels in Sulu Üzüm, may contribute to UV protection and cellular defense mechanisms (Ozturk et al. 2019). Overall, genotypes such as Sulu Üzüm, Dımışkı, and Kuru Üzüm demonstrated the most diverse and abundant phenolic profiles, highlighting their biochemical richness and potential importance for future breeding and compositional studies. In addition to Vural (2011) and R. Gazioglu Sensoy (2012) indicate that compounds such as protocatechuic acid, chlorogenic acid, and p‐hydroxybenzoic acid play critical roles in antioxidant capacity and biological activity. These findings, particularly in our study, confirm that varieties such as Sulu Üzüm and Dımışkı are valuable candidates for future biochemical and nutritional research. Similarly, Karadoğan and Keskin (Karadogan and Keskin 2017) reported that high anthocyanin and phenolic acid accumulation in black plum clones was strongly correlated with antioxidant activity. Therefore, the inter‐genotype differences in individual phenolic compounds suggest that these cultivars offer significant biochemical diversity for both consumer health and industrial applications.

### 3.8 Principal Component Analysis and Hierarchical Clustering

**FIGURE 2:**
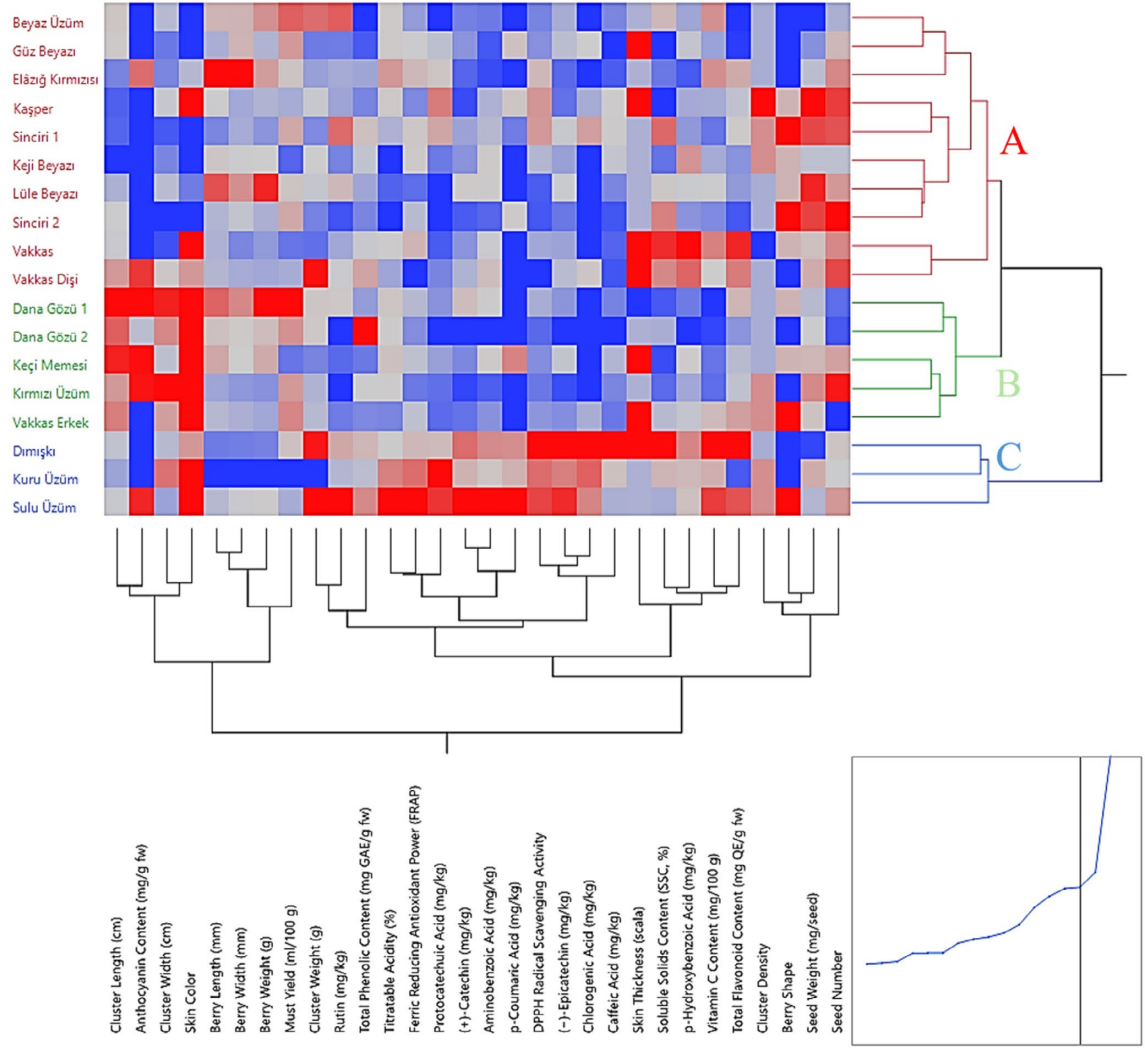
Heatmap with hierarchical clustering of 18 autochthonous grape genotypes based on pomological, biochemical, and phenolic parameters. Sample dendrogram (right) illustrates genotype similarity; feature dendrogram (bottom) shows trait associations. Color gradient indicates relative expression levels. Clusters A (red), B (green), and C (blue) represent major genotype groupings.

Multivariate analysis was performed to classify the 18 autochthonous grape genotypes based on their integrated pomological, biochemical, and phenolic profiles. The clustering history identified the closest pairwise similarity between Lüle Beyazı and Sinciri 2 (distance = 3.28), both characterized by moderate berry size and low anthocyanin content. In contrast, the most distant relationship was observed between Beyaz Üzüm and Dımışkı (distance = 11.73), reflecting their opposing biochemical profiles particularly in total phenolic content and antioxidant activity. Biochemical clustering was evident in the pairing of Dımışkı with Sulu Üzüm and Kuru Üzüm, genotypes known for elevated catechin, epicatechin, and DPPH values. Pomological clustering was observed between Vakkas and Vakkas Dişi, which share similar cluster morphology and berry dimensions. The pairing of Beyaz Üzüm and Güz Beyazı further supports the grouping of light‐skinned, table‐type genotypes with moderate juice yield and low phenolic accumulation. Trait clustering revealed strong biochemical and morphological associations. For instance, (+)‐Catechin and Aminobenzoic Acid clustered tightly (distance = 0.95), suggesting co‐accumulation in high‐antioxidant genotypes such as Dımışkı and Sulu Üzüm. Pomological traits such as Berry Length, Width, and Weight formed a coherent cluster, confirming their morphological interdependence. Antioxidant‐related traits clustered meaningfully: DPPH with Epicatechin (1.78) and Caffeic Acid (2.29), supporting the role of specific flavonoids in radical scavenging. Notably, Cluster Weight and Total Phenolic Content (3.90) formed a phenolic cluster, suggesting that heavier clusters may correlate with higher biochemical richness. Similarly, Vitamin C and Total Flavonoid Content (2.76) clustered together, indicating metabolic coordination in antioxidant biosynthesis. The pairing of Soluble Solids Content (SSC) with p‐Hydroxybenzoic Acid (2.82) implies a link between sugar accumulation and phenolic metabolism (Tables 8 and 9; Figure 2). These clustering patterns are consistent with previous studies that used PCA and heatmap‐based clustering to differentiate grape genotypes based on metabolite profiles and antioxidant capacity (Moradi et al. 2024). The integration of pomological and biochemical traits in multivariate analysis provides a robust framework for genotype classification, selection, and functional food development.

**TABLE 8:** Clustering history of grape genotypes based on Euclidean distance.

Cluster step	Distance	Leader	Joiner
Closest pair	3.28	Lüle Beyazı	Sinciri 2
Furthest pair	11.73	Beyaz Üzüm	Dımışkı
Biochemical cluster	6.41	Dımışkı	Sulu Üzüm
Biochemical cluster	6.04	Dımışkı	Kuru Üzüm
Pomological cluster	3.73	Vakkas	Vakkas Dişi
Pomological cluster	3.33	Beyaz Üzüm	Güz Beyazı

**TABLE 9:** Feature clustering of biochemical and pomological traits.

Cluster step	Distance	Leader	Joiner
Biochemical pair	0.95	Catechin	Aminobenzoic acid
Pomological pair	1.17	Berry length	Berry width
Antioxidant pair	1.78	DPPH	Epicatechin
Antioxidant pair	2.29	DPPH	Caffeic acid
Phenolic cluster	3.90	Cluster weight	Total phenolic content
Flavonoid cluster	2.76	Vitamin C	Total flavonoid content
Sugar‐acid pair	2.82	SSC	p‐hydroxybenzoic acid

### 3.9 Principal Component Analysis (PCA)

**FIGURE 3:**
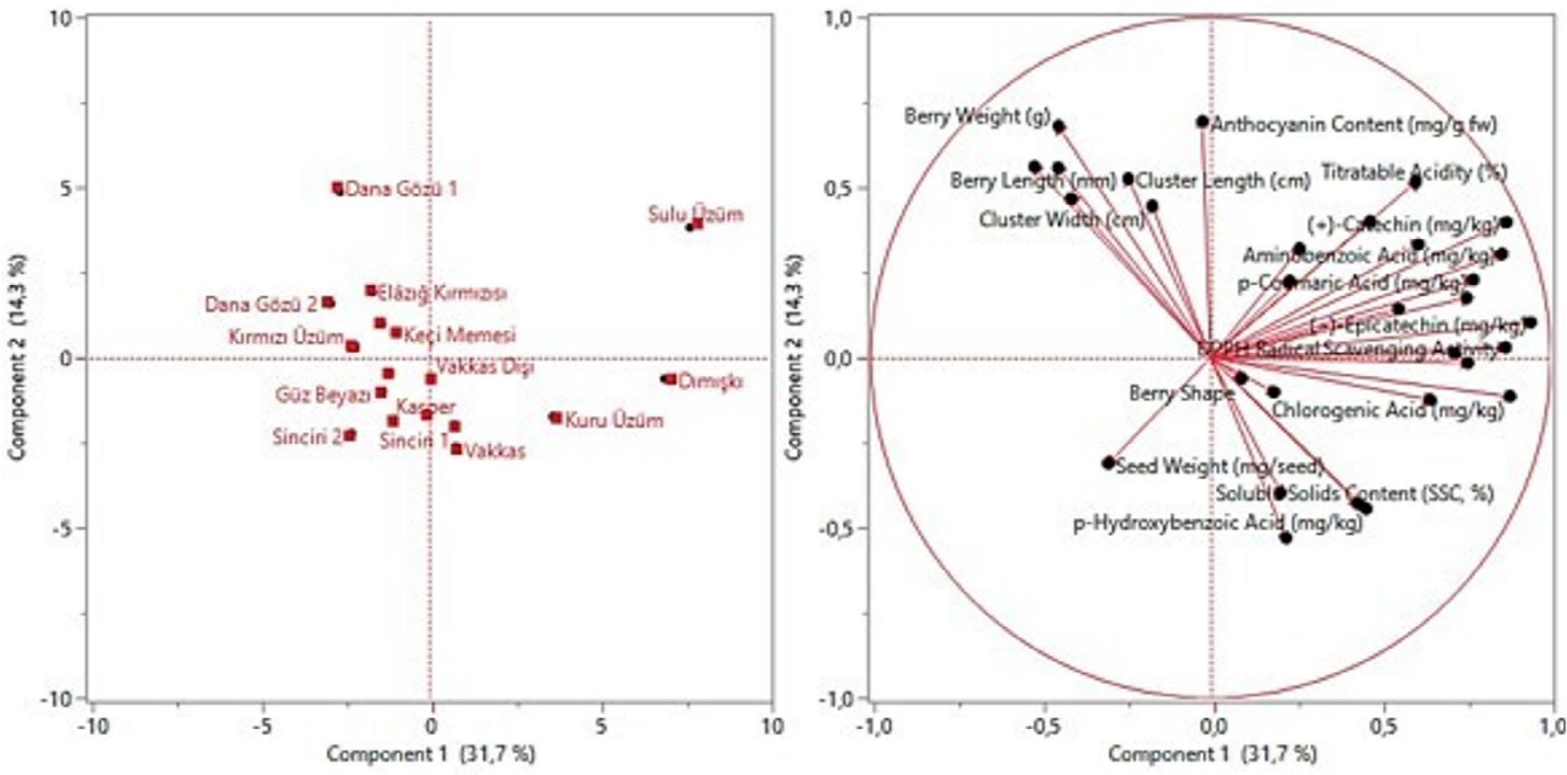
PCA biplot of grape genotypes and traits. Left panel shows genotype distribution; right panel displays trait loadings across PC1 and PC2. Red points represent genotypes; black vectors indicate trait contributions. Trait clustering reveals strong associations among antioxidant variables and morphological traits.

PCA was conducted to explore multivariate relationships among grape genotypes and their integrated pomological and biochemical traits. The first two principal components explained 51.9% of the total variance (PC1: 37.8%, PC2: 14.1%), as visualized in Figure 3 and detailed in Table 10. PC1 was primarily defined by antioxidant‐related traits, including epicatechin (loading: 0.305), catechin (0.282), DPPH (0.281), FRAP (0.244), vitamin C (0.245), and chlorogenic acid (0.285). These variables collectively define the biochemical richness axis, distinguishing genotypes with elevated antioxidant capacity. Genotypes such as Dımışkı, Sulu Üzüm, and Kuru Üzüm scored positively on PC1, reflecting their high phenolic and flavonoid content. PC2 was shaped by pomological traits, particularly berry weight (0.329), berry length (0.271), berry width (0.270), and anthocyanin content (0.336). These traits define the fruit morphology and pigmentation axis, separating genotypes based on berry size and skin color. Genotypes such as Kırmızı Üzüm, Keçi Memesi, and Vakkas Dişi were positioned positively on PC2, indicating larger berries and higher anthocyanin accumulation. Trait vectors including epicatechin, catechin, and DPPH were closely clustered in the biplot, suggesting strong intercorrelation and shared contribution to antioxidant capacity. In contrast, traits like cluster length, berry shape, and seed weight showed orthogonal orientation, indicating independence from biochemical traits and minimal influence on PC1 and PC2. These PCA results are consistent with previous grape metabolomics studies, where antioxidant traits and berry morphology were shown to drive genotype separation and functional classification (Prakash et al. 2020; Yang et al. 2022). The biplot structure supports the identification of trait‐driven genotype clusters and highlights key variables for breeding, nutritional profiling, and quality assessment. It should also be noted that the present study was conducted in a single production season under specific ecological conditions of Muş province. Environmental factors such as temperature, solar radiation, precipitation, soil properties, and vineyard management practices are known to significantly influence grape biochemical composition and antioxidant metabolism. Therefore, the observed biochemical and pomological variability among genotypes may partially reflect environmental effects in addition to genetic differences. Multi‐year and multi‐location studies would provide a more comprehensive understanding of genotype stability and environmental responsiveness.

**TABLE 10:** Eigenvalues and trait loadings for the first five principal components.

Trait	PCA1	PCA2	PCA3	PCA4	PCA5
Eigenvalue	9.5029	4.2817	3.1504	2.9238	2.0304
Percent	31.676	14.272	10.501	9.746	6.768
Cum percent	31.676	45.949	56.45	66.196	72.964
Cluster length (cm)	−0.07883	0.25412	−0.08266	0.36011	0.06672
Cluster width (cm)	−0.05604	0.21596	0.17972	0.37030	−0.23426
Cluster weight (g)	0.15222	0.19403	−0.24504	0.04958	−0.01370
Cluster density	0.06024	−0.04868	0.41482	−0.06248	−0.19298
Berry length (mm)	−0.16827	0.27112	−0.11478	−0.20418	0.18662
Berry width (mm)	−0.14564	0.26957	−0.11906	−0.29729	0.07332
Berry weight (g)	−0.14553	0.32911	−0.13129	−0.18272	0.08456
Berry shape	0.02942	−0.02903	0.13784	−0.20054	0.22461
Skin thickness (scala)	0.07154	−0.25538	−0.11520	0.18884	0.20077
Skin color	0.08442	0.15490	0.17002	0.42807	0.16130
Seed number	0.06671	−0.19165	0.06783	−0.16751	0.28427
Seed weight (mg/seed)	−0.09786	−0.15006	0.32749	0.00077	0.26798
Must yield (mL/100 g)	−0.13352	0.22605	−0.15957	−0.02814	−0.31659
Soluble solids content (SSC. %)	0.14021	−0.20664	−0.31022	0.05141	−0.01595
Titratable acidity (%)	0.19555	0.24960	0.18185	0.00137	0.05936
Vitamin C content (mg/100 g)	0.24536	−0.00736	−0.23623	−0.07934	0.03575
Total phenolic content (mg GAE/g fw)	0.07505	0.10900	0.01035	0.22532	−0.03331
Total flavonoid content (mg QE/g fw)	0.17924	0.06914	−0.18885	0.18329	0.26914
Anthocyanin content (mg/g fw)	−0.00804	0.33553	0.10148	0.17444	0.28237
DPPH radical scavenging activity	0.28134	0.01468	0.08440	−0.08384	−0.17737
Ferric reducing antioxidant power (FRAP)	0.24423	0.08514	0.15038	−0.15164	0.05918
(+)‐catechin (mg/kg)	0.28193	0.19228	−0.01241	−0.06295	0.02733
(−)‐epicatechin (mg/kg)	0.30526	0.05034	−0.07098	−0.02394	−0.08001
Rutin (mg/kg)	0.19801	0.16072	−0.08640	−0.27208	−0.03194
p‐coumaric acid (mg/kg)	0.25049	0.11098	0.13690	−0.05803	0.12710
Caffeic acid (mg/kg)	0.20946	−0.05966	−0.20098	0.08810	−0.26300
Chlorogenic acid (mg/kg)	0.28542	−0.05484	−0.01624	0.04931	−0.24347
Aminobenzoic acid (mg/kg)	0.27787	0.14783	0.00957	−0.04672	0.18335
Protocatechuic acid (mg/kg)	0.23295	0.00648	0.33357	−0.07942	−0.07643
p‐hydroxybenzoic acid (mg/kg)	0.14840	−0.21449	−0.20333	0.14504	0.31739

### 3.10 Trait Interactions Revealed by Pearson's Correlation Analysis

**FIGURE 4:**
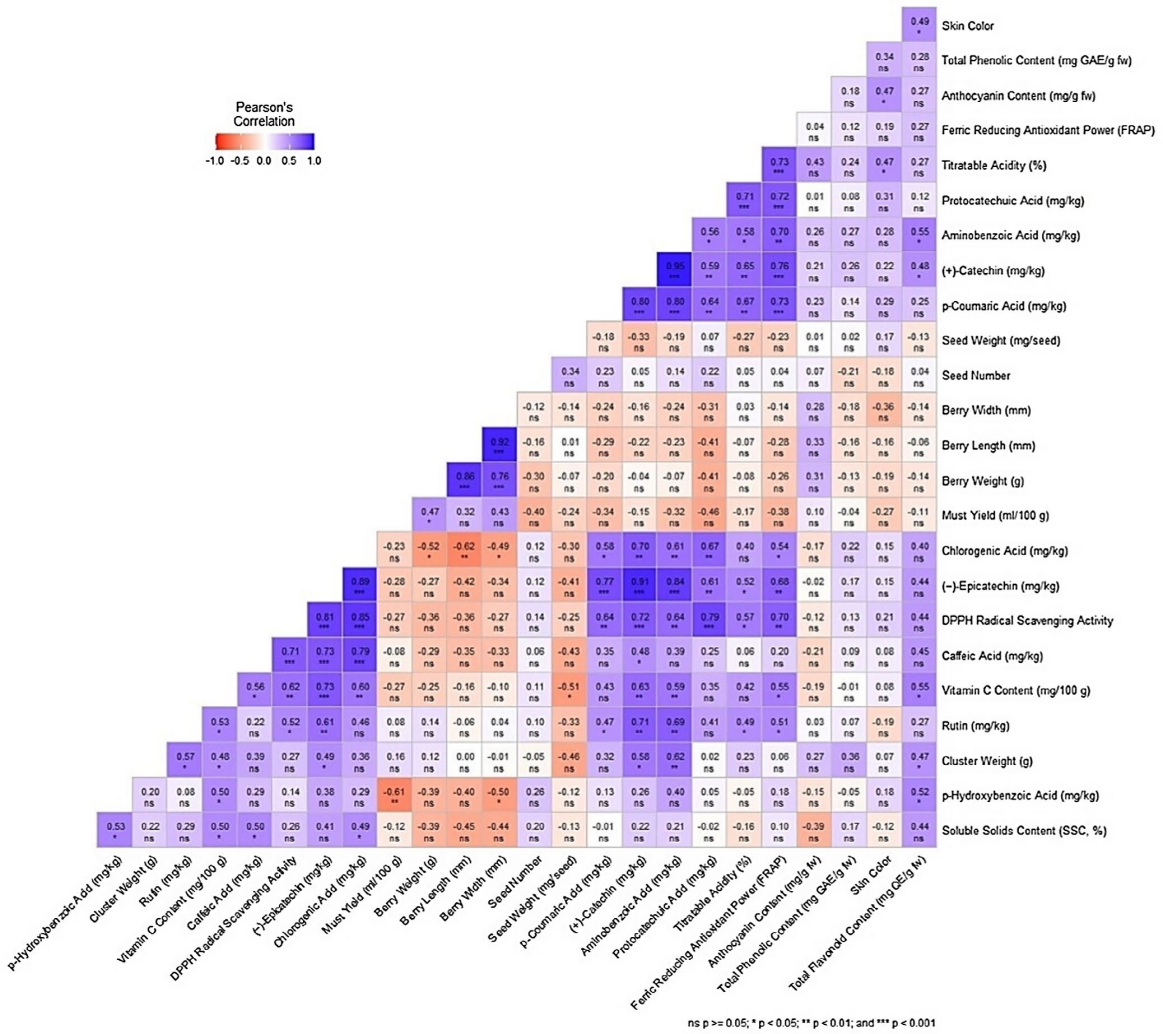
Triangular correlation matrix of pomological, biochemical, and antioxidant traits in grape genotypes. Purple shades indicate positive correlations; orange shades indicate negative correlations. Numerical values represent Pearson's *r coe*fficients; asterisks denote significance levels.

Pearson's correlation analysis was conducted to examine the relationships among pomological, biochemical, and antioxidant traits across 18 autochthonous grape genotypes. The full correlation matrix is visualized in Figure 4, where both the strength and direction of associations are indicated by color intensity. Statistical significance is denoted as *p* ≤ 0.05 (*), *p* ≤ 0.01 (**), and *p* ≤ 0.001 (***).

Antioxidant traits such as DPPH, FRAP, catechin, and epicatechin were strongly and positively correlated (*r* > 0.70, ***), confirming their biochemical synergy in radical scavenging. This supports previous findings that polyphenolic compounds act cooperatively in antioxidant defense (Milella et al. 2023). Vitamin C content showed significant positive correlations with total flavonoid content (*r* = 0.55) and titratable acidity (r* = 0.42, **), suggesting a metabolic link between organic acid accumulation and antioxidant biosynthesis (Liu et al. 2018). Anthocyanin content correlated positively with Skin Color (*r* = 0.47) and Berry Weight (r* = 0.31, **), indicating that pigmentation and berry size may co‐vary in colored genotypes. Similar trends were reported by Kamah et al. (2025) in dark‐skinned grape shoots. Pomological traits such as berry length, width, and weight were tightly intercorrelated (*r* > 0.75, ***), reflecting morphological coherence. However, these traits were negatively correlated with skin thickness and seed number, suggesting a trade‐off between berry expansion and structural density. SSC was positively correlated with Vitamin C (*r* = 0.50) and caffeic acid (r* = 0.50, ***), indicating that sugar accumulation may enhance phenolic biosynthesis during ripening. Cluster weight showed strong positive correlations with multiple antioxidant traits, including (+)‐Catechin (*r* = 0.58), Epicatechin (r* = 0.49), and Rutin (r* = 0.57, ***), suggesting that heavier clusters may be biochemically richer. Seed weight exhibited negative correlations with antioxidant traits (e.g., *r* = −0.51 with Vitamin C), implying that genotypes with heavier seeds may allocate fewer resources to antioxidant compound synthesis.

### 3.11 Interpretation and Breeding Implications

These correlations underscore the interconnected nature of grape physiology, where morphological traits influence biochemical composition and vice versa. The strong associations among antioxidant markers suggest that selection for one trait (e.g., catechin) may simultaneously enhance others (e.g., FRAP, DPPH), suggesting coordinated variation among antioxidant‐related traits. Conversely, trade‐offs between berry size and skin thickness or seed number reflect developmental constraints and resource allocation strategies. From a breeding perspective, genotypes with high cluster weight, skin color, and vitamin C may offer dual advantages in yield and antioxidant potential. The correlation matrix supports the integration of biochemical profiling into pomological selection strategies, aligning with modern grape improvement goals.

## 4 Conclusion

This study presents a comprehensive biochemical and pomological characterization of 18 autochthonous grape genotypes, integrating phenolic profiling, antioxidant capacity, and morphological traits through multivariate analysis. The results revealed substantial inter‐genotypic variation, with cultivars such as Dımışkı, Sulu Üzüm, and Kuru Üzüm exhibiting superior antioxidant profiles, while Vakkas Dişi, Keçi Memesi, and Kırmızı Üzüm demonstrated distinct pomological advantages. The strong correlations among antioxidant markers—particularly catechin, epicatechin, DPPH, and FRAP—highlight the biochemical coherence of phenolic metabolism in grapes. Simultaneously, PCA and hierarchical clustering confirmed that morphological traits such as berry weight, skin color, and cluster structure are closely linked to biochemical richness, suggesting that yield and nutritional quality can be co‐optimized. Importantly, the integration of correlation analysis with PCA provided a robust framework for identifying trait‐driven genotype clusters, offering valuable insights for breeding programs targeting grape quality improvement and cultivar preservation. The observed trade‐offs between berry expansion and seed or skin density underscore the need for balanced selection strategies that consider both agronomic performance and nutritional value. Overall, this work contributes to the modernization of grape characterization by combining traditional pomological metrics with advanced biochemical and statistical tools. It reinforces the importance of trait integration in cultivar evaluation and sets the stage for future research on genotype–environment interactions, metabolomic mapping, and targeted breeding for nutritional enhancement. Nevertheless, the antioxidant‐related findings of this study are based solely on compositional analyses and in vitro chemical assays. Therefore, the biological relevance and potential health effects of these grape genotypes should be validated through further bioaccessibility, cellular, physiological, and in vivo studies before drawing conclusions regarding nutraceutical or functional food applications.

## Author Contributions

**Yakup Karasu:** methodology, investigation, conceptualization, validation, writing. **Adnan Dogan:** methodology, investigation, conceptualization, validation, writing. **Semih Aykut:** methodology, investigation, conceptualization, validation, writing – original draft, visualization. **Burhan Ozturk:** methodology, investigation, conceptualization, validation, writing – original draft, visualization. **Erdal Aglar:** methodology, investigation, conceptualization, validation, writing – original draft, visualization.

## Funding

This study was supported by the Scientific Research Projects Directorate of Van Yüzüncü Yıl University under project number FYL‐2023‐10795. The Open Access publication fee (Article Processing Charge) is covered through the TÜBİTAK ULAKBİM Open Access Agreement with the publisher.

## Disclosure

*Author Approval and Responsibility Statement*: All authors have read and approved the final version of the manuscript. E.A., as the corresponding author, had full access to all of the data in this study and takes complete responsibility for the integrity of the data and the accuracy of the data analysis.

## Ethics Statement

This study was conducted using autochthonous grape genotypes naturally cultivated in farmers' vineyards in Muş province, Türkiye. No human participants, animals, or protected/endangered plant species were involved in the study. Verbal permissions were obtained from vineyard owners prior to sample collection. The study complied with national and institutional regulations, including Environment Law No. 2872 and Plant Variety Protection Law No. 5042. Therefore, formal ethical committee approval was not required for this research.

## Consent

The authors have nothing to report.

## Conflicts of Interest

The authors declare no conflicts of interest.

## Data Availability

The data that support the findings of this study are available on request from the corresponding author. The data are not publicly available due to privacy or ethical restrictions.
